# Inhibition of Zinc Dendrites in Zinc-Based Flow Batteries

**DOI:** 10.3389/fchem.2020.00557

**Published:** 2020-07-24

**Authors:** Leibin Guo, Hui Guo, Haili Huang, Shuo Tao, Yuanhui Cheng

**Affiliations:** ^1^College of Chemical Engineering, Beijing University of Chemical Technology, Beijing, China; ^2^School of Chemistry and Chemical Engineering, Shandong Provincial Key Laboratory of Chemical Energy Storage and Novel Cell Technology, Liaocheng University, Liaocheng, China

**Keywords:** flow battery, zinc deposition, zinc dendrites, interfaces engineering, energy storage and conversion, rechargeable battery

## Abstract

Zinc-based flow batteries have gained widespread attention and are considered to be one of the most promising large-scale energy storage devices for increasing the utilization of intermittently sustainable energy. However, the formation of zinc dendrites at anodes has seriously depressed their cycling life, security, coulombic efficiency, and charging capacity. Inhibition of zinc dendrites is thus the bottleneck to further improving the performance of zinc-based flow batteries, but it remains a major challenge. Considering recent developments, this mini review analyzes the formation mechanism and growth process of zinc dendrites and presents and summarizes the strategies for preventing zinc dendrites by regulating the interfaces between anodes and electrolytes. Four typical strategies, namely electrolyte modification, anode engineering, electric field regulation, and ion transfer control, are comprehensively highlighted. Finally, remaining challenges and promising directions are outlined and anticipated for zinc dendrites in zinc-based flow batteries.

## Introduction

Energy and environment are the foundation of human survival and development (Zhang et al., 2019a). To meet increasing requirements, people are exploring sustainable and clean energy (Turner, 1999). However, sustainable and clean energy, represented by wind, solar, and tidal, are affected by climate and cannot directly generate continuous and stable electrical power (Yang et al., 2011; Lou et al., 2020). Large-scale energy storage devices seem to be the best choice for collecting the fluctuating energy and outputting high-quality power (Dunn et al., 2011; Leadbetter and Swan, 2012).

Flow batteries have received widespread attention due to their high safety and low cost (Liu et al., 2019a; Zhang et al., 2019a; Ye et al., 2020a,b). Their power and capacity can be designed independently. The power is determined by the number and size of the stacks, while the capacity is limited by the volume and concentration of the electrolyte outside stacks. Their capacity can be easily be increased by increasing the number of redox couples in the electrolyte without adding other equipment. Therefore, flow batteries are very suitable for large-scale energy storage.

Zinc-based flow batteries (ZFBs) have the advantages of low cost, high safety, flexible structure, and high energy efficiency and have been extensively studied (Arenas et al., 2018). Various ZFBs have been proposed, such as the zinc-bromine flow battery (Jeon et al., 2014; Suresh et al., 2014), zinc-iodine flow battery (Xie et al., 2019), zinc-nickel flow battery (Cheng et al., 2014b, 2019c; Huang et al., 2018), zinc-air flow battery (Cheng et al., 2018, 2019b), zinc-iron flow battery (Yuan et al., 2018a; Chang et al., 2019), and zinc-manganese flow battery (Liu et al., 2020). Some of these flow batteries, like the zinc-bromine flow battery, zinc-nickel flow battery, zinc-air flow battery, and zinc-iron battery, are already in the demonstration stage and are close to commercial application (Arenas et al., 2018).

**Figure 1:**
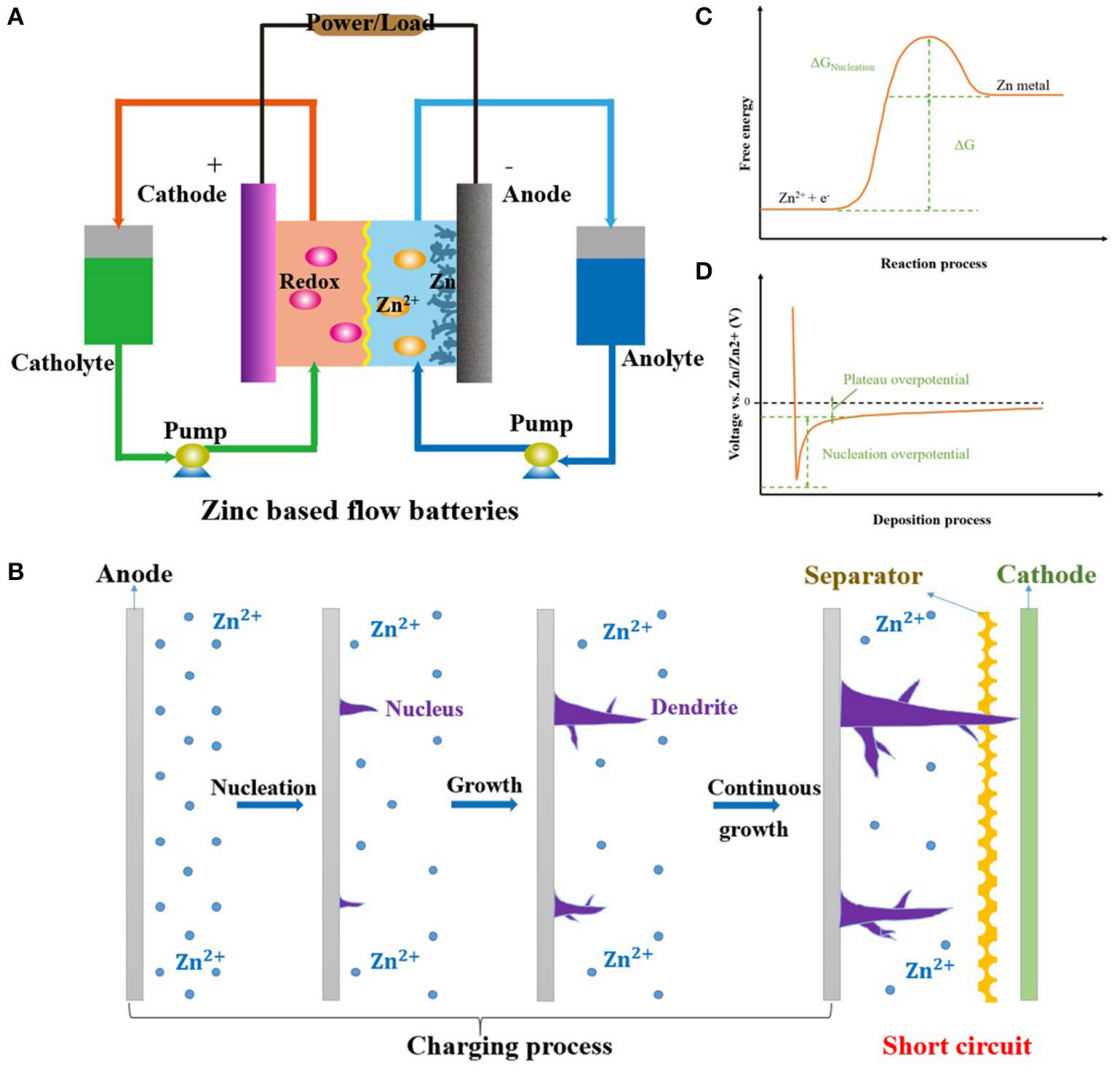
**(A)** Principle and structure of typical ZFBs. **(B)** Schematic of the nucleation and growth processes of zinc dendrites. **(C)** The energy barrier for the zinc nucleation and growth processes. **(D)** Overpotential of zinc nucleation and growth processes. Reproduced from Pei et al. (2017) with permission. Copyright 2017 American Chemical Society.

The structure and mechanism of ZFBs are shown in Figure 1A. The electrochemical reaction at the anode side is zinc deposition and stripping. This is a little different in aqueous acid/neutral and alkaline solutions (Khor et al., 2018).

1. Acid or neutral solution
  *Zn*^2+^ + 2*e*^−^ ↔ *Zn     E*^*o*^ = −0.763 *V* *vs. SHE*
2. Alkaline solution
  ${Zn\left(OH\right)}_{4}^{2-}+2e^{-}↔Zn+4OH^{-}\;E^{0}=-1.216\;V$ *vs. SHE*

However, zinc dendrites are formed during the charging process and eventually pierce the separator, resulting in short circuit and battery failure (Figure 1B). Moreover, zinc dendrites can easily fall from anodes, resulting in a decrease in efficiency and capacity (Cheng et al., 2014a). Therefore, inhibiting zinc dendrite formation is very important for the further development of ZFBs. Recently, researchers have done a lot of work to solve zinc dendrite formation through modifications to the electrolyte, anodes, electric field, and zinc ion transfer. In this review, we will introduce the formation and growth mechanism of zinc dendrites, summarize typical methods for solving zinc dendrite formation, and outline promising future directions.

## Formation Mechanism of Zinc Dendrites

Zinc deposition begins with nucleation and continues with growth (Yufit et al., 2019; Zheng et al., 2019). The energy barrier for zinc nucleation is much higher than for zinc growth on the nucleus, as shown in Figure 1C (Zeng et al., 2019; Zhang et al., 2020). As a result, the overpotential of zinc nucleation is also larger than that of zinc growth on the nucleus (Figure 1D; Zhang et al., 2019b, 2020). This indicates that once a zinc nucleus forms, zinc ions prefer to deposit on the nucleus rather than to produce a new nucleus. Moreover, small nuclei have high surface energy and thermodynamically tend to aggregate into larger particles (Pei et al., 2017; Cheng et al., 2019a). Therefore, it is very difficult to obtain uniform zinc nuclei on the anode.

During the growth process, zinc ions migrate to a nucleus under the driving forces of electric fields and concentration gradients (Wang et al., 2015; Lacitignola et al., 2017). The distributions of the electric field and zinc ions at the interface between anodes and electrolytes play an important role in zinc deposition (Cheng et al., 2013b, 2014a). A uniform electric field is favorable for both the nucleation and growth of zinc deposits. Unfortunately, the electric field is much stronger in the areas adjacent to current collectors than at edges and corners far away from current collectors (Cheng et al., 2013b). After zinc ions at the interface are consumed, zinc ions that exist in the electrolyte far from the interface cannot migrate to the interface in time, resulting in severe concentration polarization (Wang et al., 2015). Simultaneously, zinc ions preferentially migrate to the protruding tips of anodes and subsequently grow on previously deposited zinc seeds, which accelerates the formation of zinc dendrites (Lu et al., 2018). Additionally, hydrogen evolution at an anode also makes mass transfer more difficult (Ito et al., 2011a; Dundalek et al., 2017). This phenomenon is more serious in the case of the rapid deposition of zinc ions at large anodes (Cheng et al., 2015, 2019c).

## Strategies to Prevent Zinc Dendrite Formation

**Figure 2:**
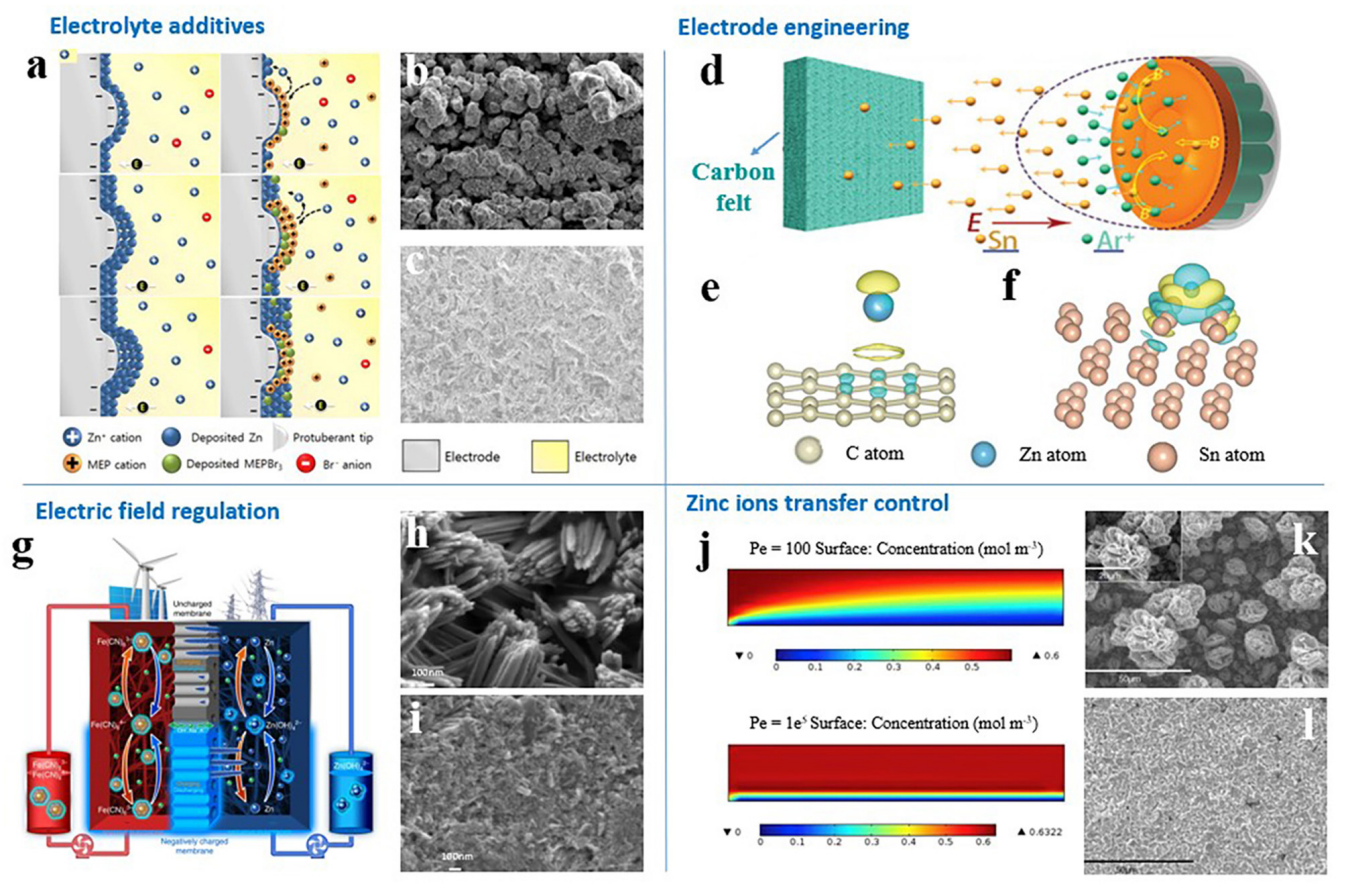
**(a)** Illustration of the prevention of zinc dendrite formation by cationic 1-Ethyl-1-methyl-pyrrolidinium bromide (MEP·Br) through an electrostatic shielding effect. **(b)** Without and **(c)** with 1.2 M MEP·Br in 2.0 M zinc-bromide electrolyte solution. X100 magnification of deposited zinc. **(a–c)** Reproduced from Kim et al. (2019) with permission. Copyright 2019 Elsevier. **(d)** Schematic illustration for the fabrication process of Sn-coated carbon felt. Interfacial charge-density of **(e)** carbon and **(f)** Sn based on DFT calculation. **(d–f)** Reproduced from Yin et al. (2020) with permission. Copyright 2020 Wiley-VCH. **(g–i)** Schematic of zinc deposition when employing an uncharged (top) and a negatively charged (bottom) membrane in a zinc–iron flow battery, and corresponding zinc morphologies at the end of charging. Reproduced from Yuan et al. (2018b) with permission. Copyright 2018 Nature Publishing Group. **(j)** Electrolyte concentration distribution at Pe = 100 and Pe = 1e^5^. Morphologies of deposited zinc in **(k)** quiescent alkaline electrolyte and **(l)** flowing alkaline electrolyte at a current density of 100 mA cm^−2^. **(j–l)** Reproduced from Wang et al. (2014) with permission. Copyright 2014 Elsevier.

Recently, various methods have been proposed to inhibit zinc dendrite formation, including electrolyte modification (Wen et al., 2012; Banik and Akolkar, 2013; Kim et al., 2019), anode engineering (Lin, 2018; Suresh et al., 2019; Yin et al., 2020), electric field regulation (Cheng et al., 2014a; Nikiforidis et al., 2014; Yuan et al., 2018b), and ion transfer control (Ito et al., 2011b; Song et al., 2014; Wang et al., 2014). In this section, we will introduce the typical solutions for preventing zinc dendrite formation in ZFBs from the above four aspects, as shown in Figure 2.

### Electrolyte Modification

Organic molecules, polymers, and metal ions are common additives for inhibiting zinc dendrites. Organic molecules and polymers can selectively adsorb onto the protruding parts of anodes and act as a barrier to the access of zinc ions (Mitha et al., 2018; Chladil et al., 2019). Therefore, they prevent zinc deposition on protruding parts and accelerate zinc nucleation and growth on dents by steric effects and/or electrostatic shielding. Compared with polymers, organic molecules have shorter chain lengths, and smaller end steric hindrances. Organic molecules are more likely to cover protruding parts and delay the deposition of zinc ions. Generally, the higher the polarity of the organic additives, the stronger the adsorption on anodes. However, excessively strong adsorption will result in severe electrochemical polarization for zinc deposition. Metal ions can affect the nucleation of zinc and thereby influence the growing process, and so, a uniform and compact zinc deposits layer can be obtained.

Organic molecules include non-ionic dimethyl sulfoxide (Hosseini et al., 2019), thiourea (Goh et al., 2014; Sun et al., 2017), diethyl ether (Xu et al., 2019), polyacrylic acid (Shimizu et al., 2019); cationic quaternary ammonium (Rossi et al., 2020), benzyl trimethyl ammonium hydroxide (Liu et al., 2019b), trimethyl octadecyl ammonium chloride (Shimizu et al., 2019), hexadecyl trimethyl ammonium bromide (Chladil et al., 2019), anionic sodium dodecyl sulfate (Miyazaki et al., 2016; Hosseini et al., 2018; Shimizu et al., 2019), and EMI-PF6 and EMI-TFSA (Song et al., 2016). Polymers include polyethyleneimine (Banik and Akolkar, 2015; Hashemi et al., 2017), Triton X-100 (Kan et al., 1998), polyvinyl alcohol (Ortiz-Aparicio et al., 2013), polyethylene glycol (Lee et al., 2006a; Ballesteros et al., 2007; Banik and Akolkar, 2013), polyacrylamide (Zhang et al., 2019b), Tween 20 (Chladil et al., 2019), and Pluronic F-127 (Hosseini et al., 2018). Metal ions comprise Pb^2+^ (Justinijanović et al., 1973; Wen et al., 2012), Sn^2+^ (Yuan et al., 2007; Kim and Shin, 2015; Yao et al., 2019), Bi^3+^ (Wang et al., 2001), In^3+^ (Leung et al., 2011), and La^3+^ (Yang et al., 2004).

For example, cationic 1-Ethyl-1-methyl-pyrrolidinium bromide was employed as an additive in electrolytes of zinc-bromine flow batteries to prevent zinc-dendrite development through forming an electrostatic shield in and around the zinc dendrite during the charging process (Figures 2a–c; Kim et al., 2019). The zinc deposits were uniform and compact, but the charging overpotential increased by 47 mV, and discharging overpotential increased by 98 mV. The cycling life of zinc-bromine flow batteries was improved by sacrificing voltage efficiency.

Tin ions promote the formation of crystal seeds and substantially improve the charge retention of the zinc-nickel flow battery. Interestingly, only a slightly negative shift in the initial potential of zinc nucleation was observed, and the rate performance and polarization properties of zinc anodes were no significantly reduced (Yao et al., 2019).

The synergy between various additives should also be noted. The synergistic effect of lead ions and TBAB can inhibit the growth of zinc dendrites, thereby obtaining smooth and dense zinc deposits in alkaline zincate electrolytes. This is beneficial for improving the cycling life of zinc-nickel flow batteries (Wen et al., 2012).

### Anode Engineering

The physicochemical properties and structure of anodes have an important effect on zinc deposition (Wei et al., 2016; Parker et al., 2017; Jiang et al., 2018). As zinc randomly deposits onto and strips from the anode, cracks are inevitable after repeated charge-discharge cycles when using pure zinc foils/sheets as anodes (Cheng et al., 2019b). To avoid rapid failure, conductive materials are usually used as a host for zinc deposition/dissolution, such as carbon (Jiang et al., 2018; Lin, 2018; Shen et al., 2018; Suresh et al., 2019; Zeng et al., 2019; Zhang et al., 2019b), nickel (Cheng et al., 2013a), copper (Zhang et al., 2019b), lead (Zhang et al., 2008), tin (Yin et al., 2020), chromium (Zhang et al., 2008), indium (Lee et al., 2006b; Nikiforidis and Daoud, 2015), and their compounds (Kang et al., 2018). Additionally, a traditional flat electrode has a low specific surface area and limits the charging current and capacity (Cheng et al., 2013b). Further design or modifications of anodes is essential to obtain uniform and compact zinc deposits and improve the performance of ZFBs (Chamoun et al., 2015; Li et al., 2015; Yan et al., 2015).

Nickel and carbon materials are widely used as anodes due to their good corrosion resistance and high electric conductivity (Li et al., 2015, 2018; Wang et al., 2016, 2017b; Xia et al., 2019). Under a large charging current, a rapid zinc deposition process occurs, which leads to severe zinc dendrite development on flat anodes because of their lower specific surface area (Xie et al., 2019). Cheng et al. for the first time, introduced three-dimensional porous nickel foam into zinc-nickel flow batteries (Cheng et al., 2013b). Its high specific surface area reduces the actual current density. Its three-dimensional porous structure greatly reduces the internal resistance of the interface between electrodes and electrolytes. Thus, zinc dendrite was prevented, and improved power density, energy efficiency, and cycling life were reported. This indicates that three-dimensional porous electrodes are more suitable for zinc deposition and dissolution under a high charging current.

Recently, Yin et al. chose the low-cost metal Sn as the morphology-inducing material for zinc deposition (Yin et al., 2020). Magnetron sputtering technology was used to enable Sn to be firmly deposited on carbon felt without binders (Figures 2d–f). Sn possesses stronger adsorption ability to zinc atoms than does carbon, which effectively strengthened the affiliation between the Sn nanoparticles and zinc deposits. Sn-modified carbon felt thus affords more robust zinc nucleation sites and induces compact and uniform zinc deposition. The Cycling life and coulombic efficiency of zinc-bromine flow batteries were significantly improved.

### Electric Field Regulation

The electric field drives zinc nucleation on anodes and the transfer of zinc ions to the interface between anodes and electrolytes. The electric field can be controlled by the charging current (Cheng et al., 2014a; Desai et al., 2014; Nikiforidis et al., 2014; Song et al., 2014), a charged separator (Yuan et al., 2018b), and a pulsed charging model (Wang et al., 2015, 2017a; Zelger et al., 2016; Garcia et al., 2017; Pichler et al., 2017; Yang et al., 2019).

A charged separator provides an effective way to solve zinc dendrite development in ZFBs. As shown in Figures 2g–i, Yuan et al. designed a porous membrane with negative charges on the pore wall and surface (Yuan et al., 2018b). The negatively charged zincate ions and the negatively charged porous membrane repel each other. Therefore, zinc ions can be deposited easily along the direction of the separator to the 3D carbon felt frame. A ZFB using a negatively charged membrane has no short circuit in about 240 cycles at current densities of 80 to 160 mA/cm^2^ and exhibits stable performance.

Ito et al. studied the effect of charging currents on zinc morphology in flowing alkaline electrolytes (Ito et al., 2012). The ratio of the effective current density to the limiting current density (current density ratio) is directly related to the zincate concentration on the interface and determines the morphology of zinc deposits. When the current density ratio is <0.4, the zinc morphology is mossy and porous. When the current density ratio is between 0.4 and 0.9, it has a mixture of a mossy and crystal structure. Only when the current density ratio is higher than 0.9 will the zinc deposits become crystalline and dense.

The charging module can be designed and operated to inhibit zinc dendrite (Wang et al., 2015; Pichler et al., 2017, 2018; Yang et al., 2019). The Taguchi method was utilized to optimize the values of current density, duty cycle, and pulse frequency. As the nucleation is mainly determined by overpotentials and zinc ion distribution on anodes, large overpotentials can produce more zinc seeds. Interestingly, pulsed current or voltage provides more time for zinc transfer to reactive interfaces. This will be prone to form compact and uniform zinc deposits and prevent zinc dendrite development in zinc-air flow batteries (Yang et al., 2019).

### Zinc Ion Transfer Control

Zinc ion transfer plays an important role in the growth of zinc on the nucleus. A uniform distribution of zinc ions will result in the same rate of zinc growth on anodes (Nikiforidis et al., 2014; Song et al., 2014). However, the concentration gradient of zinc ions may be different along the interfaces due to non-uniform zinc seeding. Accurate regulation of zinc ion transfer is needed. Currently, controlling the flow rate of electrolyte and adding extra magnetic field are two typical methods for achieving this (Shi et al., 2013; Wang et al., 2017a, 2018a,b).

Flowing electrolyte can change the mass transfer of zinc ions from diffusion control in static electrolytes to convection control (Wang et al., 2014). As shown in Figures 2j–l, the larger the flow velocity is, the greater the zinc concentration gradient is in the interfaces. The concentration gradient is the main driving force for zinc ion transfer. A large concentration gradient can ensure the timely delivery of reactants for the nucleation and growth process of zinc deposition. Therefore, zinc dendrites appear under quiescent electrolyte, while uniform and compact zinc deposits are obtained in flowing electrolyte (Wang et al., 2014).

Ito et al. also found that when the flow rate of the electrolyte is higher than 15 cm/s, the growth of zinc dendrites is deformed in the direction of the electrolyte flow, thereby avoiding short circuit of the battery. A zinc-nickel system with a 100 Wh battery was scaled up to evaluate the influence of zinc ion transfer on zinc morphology and battery performance. This system had a long cycling life of more than 200 cycles (Ito et al., 2011b).

A magnetic field can affect the movement of zinc ions (Wang et al., 2018a). The magnet is placed on the anode side to design an additional driving force for zinc ion transfer. The magnetic field accelerates zinc ion transfer and suppresses the dendritic growth of zinc deposits. As a result, the cycling life of batteries is improved.

## Summary and Outlook

Zinc anodes are usually used in aqueous electrolytes, enabling zinc-based batteries with high safety and low cost. Flowing electrolyte can enhance mass transfer and reduce concentration polarization. ZFBs have therefore been investigated widely and show prospects for practical application. The issue of zinc dendrite formation has been extensively studied since its emergence. Some effective strategies for inhibiting zinc dendrite development in ZFBs have been proposed, including electrolyte modification, anode engineering, electric field regulation, and ion transfer control. Although great progress has been made in the field of zinc dendrites, many methods are used in isolation, with strict working conditions, and costly implementation. Here, remaining challenges and promising directions for the inhibition of zinc dendrite formation in ZFBs are outlined and anticipated.

i) The forms of zinc ions existing in aqueous solution are very complex. Zinc ions can combine with different amounts of water and other anions, which has a significant impact on the nucleation and growth processes of zinc deposition.

ii) Zinc morphology depends strongly on specific operating conditions, such as the charging model, current density, flow rate, zinc ion concentration, and temperature. Most current studies only investigate one or two variables, idly fixing other parameters. It is necessary to systematically investigate the relationship between zinc morphologies and operating conditions. The theoretical basis of our understanding of zinc deposition needs to be enriched.

iii) The capacity and thickness of zinc deposits in ZFBs is much larger than that of lithium deposits in lithium batteries. The zinc deposits in ZFBs are expected to have a specific capacity of more than 100 mAh/cm^−2^ and to be thicker than 170 μm. The thicker the zinc deposit is, the more difficult it is to control its morphology. Therefore, great efforts are required to concentrate on the inhibition of zinc dendrites under large capacity or thick deposits.

iv) In static zinc batteries, brighteners, pretreatment of zinc anodes, and new electrolytes have made significant progress toward achieving a uniform zinc electroplating/electrostripping process, which may enable flow battery researchers to look into more possibilities in further work.

In short, we look forward to a better solution to the zinc dendrite problem with a view to achieving a long cycling life and high safety and eventually improving the competitiveness of ZFBs.

## Author Contributions

All authors listed have made a substantial, direct and intellectual contribution to the work, and approved it for publication.

## Conflict of Interest

The authors declare that the research was conducted in the absence of any commercial or financial relationships that could be construed as a potential conflict of interest.
